# Severe Thrombocytopenia, Thrombosis and Anti-PF4 Antibody after Pfizer-BioNTech COVID-19 mRNA Vaccine Booster—Is It Vaccine-Induced Immune Thrombotic Thrombocytopenia?

**DOI:** 10.3390/vaccines10122023

**Published:** 2022-11-26

**Authors:** Victor W. T. Ling, Bingwen Eugene Fan, Soon Lee Lau, Xiu Hue Lee, Chuen Wen Tan, Shir Ying Lee

**Affiliations:** 1 Department of Haematology-Oncology, National University Hospital, Singapore 119074, Singapore; 2 Department of Haematology, Tan Tock Seng Hospital, Singapore 308433, Singapore; 3 Lee Kong Chian School of Medicine, Nanyang Technological University, Singapore 308232, Singapore; 4 Department of Haematology, Singapore General Hospital, Singapore 169608, Singapore; 5 Division of Haematology, Department of Laboratory Medicine, National University Hospital, Singapore 119074, Singapore

**Keywords:** COVID-19 vaccine, VITT, thrombocytopenia, thrombosis, anti-PF4 antibody, lupus anticoagulant

## Abstract

Vaccine-induced immune thrombotic thrombocytopenia (VITT) is a serious and life-threatening complication occurring after adenovirus-vector COVID-19 vaccines, and is rarely reported after other vaccine types. Herein, we report a case of possible VITT after the Pfizer-BioNTech mRNA vaccine booster, who presented with extensive lower limb deep vein thrombosis, severe thrombocytopenia, markedly elevated D-dimer and positive anti-PF4 antibody occurring 2 weeks post-vaccination, concurrent with a lupus anticoagulant. A complete recovery was made after intravenous immunoglobulin, prednisolone and anticoagulation with the oral direct Xa inhibitor rivaroxaban. The presenting features of VITT may overlap with those of antiphospholipid syndrome associated with anti-PF4 and immune thrombocytopenia. We discuss the diagnostic considerations in VITT and highlight the challenges of performing VITT confirmatory assays in non-specialized settings. The set of five diagnostic criteria for VITT is a useful tool for guiding initial management, but may potentially include patients without VITT. The bleeding risks of severe thrombocytopenia in the face of thrombosis, requiring anticoagulant therapy, present a clinical challenge, but early recognition and management can potentially lead to favorable outcomes.

## 1. Introduction

Since the start of the COVID-19 pandemic, vaccines against the SARS-CoV-2 virus have greatly reduced symptomatic and severe disease, hospitalizations and deaths due to COVID-19 infection [1,2,3,4]. Clinical trials of COVID-19 vaccines showed them to be highly efficacious and safe, but due to the possibility of rare adverse vaccine side effects not observed during vaccine development, surveillance and safety monitoring after implementation of vaccination programs are still required [5].

Immune idiopathic thrombocytopenic purpura (ITP) and vaccine-induced immune thrombotic thrombocytopenia (VITT) are two rare hematologic thrombocytopenic conditions occurring after COVID-19 vaccines. Post-vaccination ITP after mRNA vaccines was reported soon after the mass roll-out of SARS-CoV-2 vaccines [6]. A review of cases reported to the United States Vaccine Adverse Events Reporting System described ITP occurring within two weeks post-vaccination, with the majority lacking pre-existing autoimmune conditions and responding to corticosteroids and intravenous immunoglobin [7,8]. The incidence of thrombocytopenia after Pfizer-BioNTech and Moderna mRNA vaccines was estimated to be 0.80 per million doses, which did not pose a safety signal compared with the baseline incidence of 3.3 ITP cases per 100,000 adults annually in the United States [9]. In contrast, the incidence of post-vaccine ITP was consistently higher with the AstraZeneca adenovirus-based ChAdOx1 vaccine [8,10,11]. Data from the Australian state of Victoria reported an incidence of 8 per million doses for ChAdOx1, twice the expected background rate of 4.1 per million, compared to the rate after the Pfizer-BioNTech vaccine, which was similar to the expected background rate. Data from Scotland, on the other hand, estimated an incidence of 1.13 cases per 100,000 doses for the AstraZeneca vaccine, but no increase associated with the Pfizer-BioNTech vaccine [10,11].

VITT is mechanistically different from ITP. VITT is a clinical syndrome caused by activating antibodies towards platelet factor 4 (anti-PF4), which is induced by SARS-CoV-2 vaccines. VITT is a recently described, rare and potentially fatal syndrome characterized by mild to severe thrombocytopenia and a striking hypercoagulable state. It leads to widespread venous or arterial thrombosis, particularly at unusual sites, such as cerebral venous sinus thrombosis and splanchnic thrombosis. VITT is closely related to the well-characterized condition termed heparin-induced thrombocytopenia (HIT). The hallmark of VITT is a positive enzyme-linked immunosorbent assay (ELISA) for anti-PF4 [12]. Vaccine components are thought to form antigenic complexes with PF4 on platelet surfaces, triggering formation of autoantibodies targeting PF4-polyanion complexes, which can potently activate platelets and lead to platelet consumption and a prothrombotic state [13]. The prevailing diagnostic criteria proposed by experts indicate that a diagnosis of VITT should be made if all five of the following are met: (i) venous or arterial thrombosis, (ii) thrombocytopenia, (iii) occurring between 4 to 30 or 42 days after receiving the COVID-19 vaccine, (iv) presence of antibodies against PF4, and (v) markedly elevated D-dimer [14,15].

VITT was first reported in February 2021, and is strongly associated with adenovirus-based AstraZeneca ChAdOx1 and the Johnson & Johnson Ad26.COV2 vaccines [16,17,18]. The estimated incidence of VITT is 7–10 cases per million individuals for the AstraZeneca vaccine and 3.2 cases per million for the Johnson & Johnson vaccine [19]. The condition is thought to be a class effect, but recent reports of its rare occurrence after the Moderna mRNA-1273 vaccine [20,21] and human papillomavirus vaccine [22] have emerged. One probable case of VITT has also been reported after the first dose of Pfizer-BioNTech mRNA vaccine [23]. On the other hand, whether or not the incidence of thrombosis without thrombocytopenia are increased after COVID-19 vaccines is a matter of debate. There appears to be a slight positive signal for cerebral venous sinus without thrombocytopenia after COVID-19 vaccines, but thrombosis of the usual anatomical sites, i.e., deep vein thrombosis and pulmonary embolism, do not appear to be significantly increased [24].

Herein, we report a case of a young woman who developed a combination of severe thrombocytopenia and extensive venous thrombosis of the inferior vena cava and lower limb, concurrent with detectable anti-PF4 antibody and a lupus anticoagulant, occurring 2 weeks after a booster dose of the Pfizer-BioNTech COVID-19 mRNA vaccine tozinameran, BNT162b2. We also discuss the diagnostic and management considerations of the case.

## 2. Case Presentation

### 2.1. Clinical Presentation

A 27-year-old woman who was previously well presented with progressively worsening left lower limb swelling and pain, with a duration of one week. One week prior to symptom onset, she had received a COVID-19 booster dose of the Pfizer-BioNTech COVID-19 mRNA vaccine tozinameran, or BNT162b2. Her primary vaccination series of the first and second doses, using the same vaccine 5 months earlier, had been uneventful. The lower limb swelling was not preceded by any known common risk factors for venous thrombosis. Her presenting history was negative for symptoms of autoimmune disease, and she had no chest pain, shortness of breath or headache. She was not on any regular medications; in particular, she used no hormonal contraceptive or recreational drugs. Her past medical history was insignificant with regard to personal or family history of thrombosis and bleeding disorders. There were no previous blood tests, as she had no past medical history of note.

On examination, she was afebrile and hemodynamically stable. Her left leg was significantly swollen compared to the right, and tense and warm to the touch, suggestive of deep vein thrombosis. Systemic examination was unremarkable. Specifically, there were no signs of autoimmune disease and no palpable abdominal mass, and no petechiae, purpura or ecchymoses were visible.

### 2.2. Laboratory and Radiology Investigations

Full blood count revealed severe thrombocytopenia with a platelet count of 10 × 10^9^/L, mild leukocytosis (white cell count 12.5 × 10^9^/L, predominantly neutrophils) and mild microcytic hypochromic anemia (hemoglobin 11.2 g/dL, mean corpuscular volume 71 fL). Pregnancy, COVID-19 infection and dengue infection were ruled out. Serum D-dimer concentration was markedly elevated at 9050 mcg/L FEU (Reference < 500 mcg/L FEU). A venous doppler ultrasound of the left lower limb confirmed extensive deep vein thrombosis involving the left distal external iliac, great saphenous, common and superficial femoral veins and popliteal vein, with possible extension to the inferior vena cava. Review of the blood film confirmed true thrombocytopenia with occasional large platelets observed morphologically, and did not show features of autoimmune or microangiopathic hemolysis. Prothrombin Time (PT) and activated Partial Thromboplastin Time (aPTT) were not prolonged, while Clauss fibrinogen was normal at 3.97 g/L. Kidney function, liver function, serum vitamin B12 and folate were normal, and viral markers for hepatitis B, hepatitis C and human immunodeficiency virus were non-reactive. The iron indices showed low transferrin saturation (7%), low serum iron (4.1 micromol/L), normal transferrin (224 mg/dL) and normal ferritin (73 microg/L) in the presence of elevated C-reactive protein of 152 mg/L (Reference 0–10 mg/L), compatible with iron deficiency in the presence of inflammation.

Due to the occurrence of thrombocytopenia with extensive venous thrombosis, VITT was suspected, and an ELISA test for anti-platelet factor 4 (PF4) antibody was performed using the Lifecodes^®^ PF4 IgG ELISA assay (Immucor, GTI Diagnostics, Waukesha, WI, USA. Lot number 3011052). This assay detects IgG antibodies targeted against PF4 complexed with the linear polyanionic compound polyvinyl sulfonate (PVS). The result was positive, with mean optical density (OD) reactivity of 0.922 (Reference < 0.4). A confirmatory step, using an excess of heparin to confirm the heparin-dependency of the antibody, was performed according to manufacturer’s instructions. Addition of excess heparin led to a 78% inhibition of the reactivity of the patient’s sample. By this assay, inhibition of a positive reaction by >50% is considered confirmatory of the presence of specific antibodies that react with PF4-polyanion.

A second anti-PF4/HIT ELISA assay, using the Asserachrom^®^ HPIA-IgG (Diagnostica Stago, Asnières-sur-Seine, France. Lot number 259420), similarly showed positive reactivity with an OD of 0.4566 (Reference < 0.2). While ELISA assays are sensitive for anti-PF4 antibodies, not all antibodies activate platelets, and only platelet-activating antibodies are considered pathogenic in VITT.

We further proceeded to a functional assay for platelet-activating antibodies by the standard Heparin-induced Platelet Activation Assay (HIPA). The standard HIPA assay is designed to detect classic HIT antibodies. It detects platelet activation, which occurs when heparin-PF4 complex and anti-heparin-PF4 IgG antibodies combine to form an immune complex, which in turn binds to platelet Fc receptors in order to activate platelets. The patient’s serum is combined with donor-derived washed platelets, and heparin is added. In cases of classic HIT, IgG antibodies bind to heparin-PF4 complexes in the presence of low dose (0.1–0.5 U/mL) unfractionated heparin, resulting in the formation of an immune complex and, thereby, platelet activation. The resultant platelet aggregation is detected by light transmission aggregometry. The heparin-PF4 complex is, however, disrupted by high doses of heparin (100 U/mL), resulting in no platelet aggregation despite the presence of anti-heparin-PF4 antibodies. The patient’s standard washed platelet HIPA showed a lack of aggregation at low and high doses of heparin by manual visual detection of aggregation; hence, it was considered negative, but we further discuss the significance and limitations of this in the discussion. Lupus anticoagulant testing by dilute-Russell Viper Venom time (dRVVT) on the Sysmex^®^ CS-5100 platform (Sysmex, Kobe, Japan), performed prior to initiation of treatment, later returned positive. Anti-cardiolipin and anti-beta2 glycoprotein-I IgM and IgG, anti-nuclear and anti-double stranded-DNA antibodies, direct Coombs’ test and Factor V Leiden gene mutation were negative.

### 2.3. Treatment and Progress

Based on the clinical presentation and results, the possibility of VITT was considered. Autoimmune thrombocytopenia and thrombosis related to antiphospholipid antibodies was a possible differential diagnosis. The patient was immediately started on a high dose of IVIg (0.7 g/kg/day for 2 days) and corticosteroids (oral Prednisolone 1 mg/kg/day) to increase the platelet count. On day 2 post IVIg, the platelet count showed a prompt increment to 21 × 10^9^/L, and oral direct factor Xa inhibitor Rivaroxaban was started at half the therapeutic dose (15 mg daily). The choice of a non-heparin anticoagulant was made due to the concern of VITT. On day 7 post IVIg, the platelet count surpassed 50 × 10^9^/L, and Rivaroxaban was further increased to the full therapeutic dose of 15 mg twice daily. The platelet count showed a steady increase, and surpassed 100 × 10^9^/L within 2 weeks of treatment. The patient tolerated Rivaroxaban well, without bleeding complications. The symptoms of leg swelling improved tremendously and resolved by 1 month of treatment. At 2 months of treatment, the patient remained well, with a normal platelet count, and remained on therapeutic doses of Rivaroxaban and tailing doses of Prednisolone. Table 1 summarizes the clinical findings and treatment response.

## 3. Discussion

The occurrence of both severe thrombocytopenia and extensive venous thrombosis in the presence of detectable antibodies against anti-PF4, as well as marked elevation of D-dimer occurring within 30 days of vaccination, fulfills all five diagnostic criteria for VITT [14,25]. Despite this, we were unable to demonstrate platelet activating activity of the antibody using the HIPA assay. Practically, functional platelet activation assays are technically difficult and restricted only to reference labs, which precludes their timely availability to guide initial management decisions. Due to the urgent need to initiate treatment, the HIPA assay, in our case, could only be performed 1 day after IVIg, which could have induced false negative results. Due to the limitations of functional assays, which will be discussed, we believe that our case presents a case of possible VITT after the Pfizer-BioNTech vaccine booster.

VITT is closely related to the syndrome of heparin-induced thrombocytopenia (HIT). In HIT, autoantibodies against heparin-PF4 complexes form after exposure to heparin and activate platelets, leading to thrombocytopenia and widespread thrombosis. Another condition related to HIT, but without heparin exposure, has been described after infection or orthopedic surgery [26,27]. In VITT, anti-PF4 antibody has been shown to stimulate platelet activation by cross-linking platelet FcγRIIa receptors, a mechanism similar to that of HIT [16,28]. The epitope to which VITT antibody binds is located within the heparin-binding site on PF4, providing an explanation for the observation that VITT anti-PF4 antibodies are inhibited by heparin rather than enhanced by it, as seen in HIT [16,28].

In the case series from the United Kingdom [25], VITT patients after the Astra-Zeneca vaccine were generally young, with no gender predilection. They typically presented within 30 days of vaccination, with a median of 14 days, and most often after the first dose. Less commonly, isolated deep vein thrombosis or pulmonary embolism occurred up to 6 weeks after vaccination. Thrombosis was often extensive and involved several vascular beds, or both arterial and venous circulations. A high mortality rate of up to a third was observed in the initial patients, and severe thrombocytopenia and intracranial hemorrhage were associated with the highest mortality of 73%. Other clinical characteristics in VITT include marked elevation of D-dimer, a marker of intravascular coagulation activation, and low fibrinogen levels, attributed to intense consumptive coagulopathy. Our case differed from the classic presentation, as thrombosis only occurred after the booster dose, but we cannot exclude the possibility that the first dose had led to a mild chronic hypercoagulable state, which aggravated after the booster.

Diagnostic criteria for VITT have been issued to assist in the early recognition of the syndrome. The American Society of Hematology (ASH) recommends that definitive diagnosis be made if all five of the following criteria are met: (i) COVID-19 vaccine 4 to 42 days prior to symptom onset, (ii) any venous or arterial thrombosis, (iii) thrombocytopenia (platelet count < 150 × 10^9^/L), (iv) positive PF4 HIT ELISA and (v) markedly elevated D-dimer (>4 times upper limit of normal) [14]. The criteria do not define the type of vaccine. The British Society of Haematology, guidance [15], recommends a definite diagnosis of VITT be made in the presence of all five criteria: (i) onset of symptoms 5–30 days post-COVID-19 vaccine (or up to 42 days if isolated DVT/PE), (ii) presence of thrombosis, (iii) thrombocytopenia (platelet count < 150 × 10^9^/L), (iv) D dimer > 4000 mcg/L (FEU) and (v) positive anti-PF4 Antibody ELISA assay. It also makes mention of the fact that suspected VITT has been reported after Pfizer COVID-19 vaccination, although this is much more unusual. The International Society of Thrombosis and Haemostasis (ISTH) issued interim guidelines on 22 April 2021 [29] which refer specifically to adenovirus-based vaccines, as did the WHO’s guidance. A recent published review recommended a definite diagnosis upon meeting all of five criteria: (i) onset of symptoms 5–30 days post-COVID-19 vaccine (or up to 42 days if isolated DVT/PE), (ii) documented thrombosis or severe and persistent headache, (iii) thrombocytopenia (platelet count < 150,000/μL), (iv) D-dimer > 4000 FEU (and >8× ULN) and (v) positive anti-PF4/heparin IgG ELISA assay [30]. These guidelines do not specify the level of reactivity of the ELISA assay necessary to make a diagnosis of VITT, though most experts agree that higher levels of reactivity correlate more strongly with the likelihood of a positive confirmatory platelet activation assay. Among the guidelines, only the ISTH recommends testing with functional platelet activation assay. Cases with a positive anti-PF4 ELISA and a positive functional assay are confirmed to have VITT, whereas a negative functional assay would downgrade the diagnosis to “unlikely VITT;” however, they do not specify whether heparin can be resumed in these cases [29].

The mainstay of screening for HIT and VITT antibodies is the ELISA assay. ELISA assays for anti-PF4, which typically use PF4-polyanion complexes as targets, are sensitive for the purpose of detecting of VITT antibodies, whereas rapid anti-PF4 assays, such as the latex immunoturbidometric assays and chemiluminescent assays, are insensitive [19,29]. Most case series reported a high level positivity of anti-PF4 ELISA in VITT (OD > 2). A more objective analysis was performed recently in a large Australian cohort [31]. This study showed that the median OD was higher in cases confirmed by functional assay than in cases with a negative functional assay, but no specific OD cutoff level could be identified. It was noteworthy that up to a third of confirmed VITT cases presented with negative ELISA or low OD positivity [31].

Positive ELISA assays, particularly those with low OD, should ideally be followed up with a functional platelet activation assay, but such assays are not widely available. There are limitations to functional assays, as well as potential for false negatives due to several factors, such as variation of methodologies, lack of standardization, need for modification from standard assays used for HIT and inhibition of platelet reactivity by IVIg administration [30]. For example, in the case series of VITT after the Johnson & Johnson Ad26.COV2 vaccine, only one of nine VITT patients had a positive functional assay. In the United States, this is most often performed by the serotonin release assay [32]. In VITT, functional platelet activation assays are enhanced by the addition of PF4 [16,19]. It is postulated that VITT antibodies show PF4-dependent activity and may be inhibited by heparin, as opposed to classic HIT antibodies, which are augmented by heparin. This may lead to false negative functional assays designed for classic HIT. Therefore, a modification of the functional assay by addition of PF4 would be needed in order to reduce false negatives [33].

Complicating the diagnosis of VITT is the observation that low level anti-PF4 antibodies in asymptomatic individuals are common after SARS-CoV-2 vaccination. In one study, anti-PF4 occurred in 5.6% post-BNT162b2 and 8.0% post-ChAdOx1 vaccinations, typically of low titer, while in a second study in healthcare workers from Norway, the rate of anti-PF4 was 1.2% [34,35]. Most of these low-titer anti-PF4 antibodies are non-platelet activating and of little clinical significance. As a result, most experts recommend further functional platelet activation testing be performed where possible to confirm a diagnosis of VITT [29].

The management of VITT differs considerably from standard treatment of thrombosis, and, therefore, early recognition is essential. It is recommended that non-heparin anticoagulants be used, and platelet transfusions be avoided unless life-threatening bleeding occurs. The treatment of choice is administration of IVIg, which blocks platelet activation and systemic anticoagulation using non-heparin anticoagulants [14,15,29,30,36]. Anticoagulants which are considered safe are the oral and parenteral direct thrombin inhibitors, oral direct factor Xa inhibitors and fondaparinux [30]. Heparin and low molecular weight heparin should generally be avoided, largely from a cautious approach, considering the pathogenesis, potential for cross reactivity of VITT antibodies with PF4-heparin complexes and historical observation of deterioration in patients treated with heparin-based anticoagulants. However, it has not been conclusively confirmed that heparin exacerbates VITT; therefore, if non-heparin anticoagulants are unavailable, expert opinion suggests the use of heparin rather than delaying anticoagulation in this intensely prothrombotic state [30]. Corticosteroids has also been recommended, and plasma exchange has been used successfully in seriously ill patients [25]. Severe thrombocytopenia may appear to prevent anticoagulation, but experts recommend concurrent administration of IVIg to raise the platelet count in conjunction with anticoagulation [30].

In our patient, a lupus anticoagulant, a class of antiphospholipid (aPL) antibodies, was also detected. Vaccine-induced aPL antibodies and lupus anticoagulant after COVID-19 vaccines are not well-characterized. A case series described four patients who developed venous thromboembolism 2 to 3 weeks after Pfizer mRNA COVID-19 vaccination in conjunction with detectable aPL antibodies. The patients were positive for lupus anticoagulant, but negative for anticardiolipin antibodies and anti-beta2 glycoprotein I [37]. The incidence of transient lupus anticoagulant after vaccination is unknown, in contrast to an incidence of 40–50% during COVID-19 infection [38,39]. Outside of the COVID-19 and vaccination settings, a small percentage of aPL-positive individuals will have associated anti-PF4 antibodies that are non-platelet-activating. One study investigated the effect of COVID-19 vaccines on patients with known aPL antibodies pre-vaccination. Vaccination, mostly with Pfizer-BioNTech Comirnaty, did not increase the titers of pre-existing anti-PF4 antibodies, induce platelet activating ability in vitro nor trigger de novo production of anti-PF4 antibodies [40]. APL antibodies confer a prothrombotic state, and the presence of lupus anticoagulant may have posed an additional risk factor for thrombosis in this patient. In our patient, the antiphospholipid syndrome, in association with anti-PF4 and ITP post-vaccination, was an alternative diagnosis with overlapping clinical features with VITT.

## 4. Conclusions

We described a case of possible VITT in a patient after Pfizer-BioNTech mRNA COVID-19 vaccine booster. In our patient, VITT was a valid consideration, as all five diagnostic criteria for definite VITT were met. However, an alternative diagnosis of post-vaccine ITP, concurrent with a pro-coagulant state from antiphospholipid syndrome with concurrent anti-PF4 antibodies, was equally likely. This case highlights the fact that these two conditions share common presenting features, and that diagnosis of thrombocytopenia and thrombosis after COVID-19 vaccine can be challenging due to the need for specialized functional platelet activation assays, which are difficult to perform in non-specialized settings. While there remains controversy over the clinical significance of a low-titer anti-PF4, due the limited access to functional assays, we propose that patients with a positive anti-PF4, markedly elevated D-dimer, thrombocytopenia and thrombosis within the time frame of VITT, regardless of the type of vaccine, be treated with the same urgency and consideration as cases of confirmed VITT. Avoidance of heparin and low molecular weight heparin should also be strongly considered.

These results taken together, we postulate that VITT is likely to be a rare vaccine-associated adverse reaction, not limited to a class effect by modified adenovirus vector vaccines, and can also occur after other vaccines. The bleeding risks of severe thrombocytopenia in the face of thrombosis requiring anticoagulant therapy presents a clinical challenge, but early recognition and management can potentially result in favorable outcomes.

## Figures and Tables

**Table 1:** Results of platelet count, anti-PF4 HIT ELISA and standard HIPA, and a summary of the treatment response.

Day	Platelet Count (×10^9^/L)	Anti-PF4 HIT ELISA	Standard HIPA	IVIg	Corticosteroids (Prednisolone)	Anticoagulation
D1	10	Positive—OD 0.922 (Ref < 0.4) *		0.7 g/kg		
D2	21	Positive—OD 0.4566 (Ref < 0.2) ^	Negative	0.7 g/kg	1 mg/kg/day (equivalent to 90 mg daily)	Rivaroxaban 15 mg once daily
D3	40					
D7	77					Rivaroxaban 15 mg twice daily
D10	101	Positive—OD 0.717 (Ref < 0.4) *				
D17	132				80 mg daily	
D27	153				60 mg daily	Rivaroxaban 20 mg once daily
D38	198				40 mg daily	

Abbreviations: HIT = heparin induced thrombocytopenia; HIPA = Heparin induced platelet activation assay; IVIg = intravenous immunoglobulin; OD = optical density; Ref = reference range. * Lifecodes^®^ PF4 IgG. ^ Asserachrom^®^ HPIA-IgG.

## Data Availability

Not applicable.
